# Ourselves

**Published:** 1855-02

**Authors:** 


*Ourselves.*—We are almost daily in the receipt of letters from distant states, requesting specimen numbers of the Journal, or a prospectus giving the terms of subscription, etc. In reply to these letters it will be our rule to send a single number, but not to enter the name on the mail book until a subscription is formally made. Our circulation is rapidly increasing, both at home and abroad. Our special diocese is Western New York and its adjoinings, but a very large proportion of our subscribers are from the far West and South. It is a matter of regret with us that we do not receive more communications from those sources. We do not promise to publish all that we receive, but we endeavor to judge the merits of an article, not by its literary ability, but by its professional interest. A larger supply of original matter from the hundreds of competent writers on our list, would save our readers the monotony of that ever-present. H.

